# Counseling strategies and challenges for addressing infertility and infertility concerns following female genital fistula repair: perspectives from fistula care providers in Uganda

**DOI:** 10.1186/s12905-026-04388-0

**Published:** 2026-03-16

**Authors:** Suvarna Jyothi Kantipudi, Umar Senoga, Hadija Nalubwama, Justus K. Barageine, Abner P. Korn, Alison M. El Ayadi

**Affiliations:** 1 Department of Psychiatry, SRMC & RI, Sri Ramachandra Institute of Higher Education and Research(SRIHER), Chennai, India; 2 Department of Disease Control and Environmental Health, School of Public Health, College of Health Sciences, Makerere University, Kampala, Uganda; 3 Department of Obstetrics and Gynaecology, College of Health Sciences, Makerere University, Kampala, Uganda; 4 Department of Obstetrics and Gynaecology, Kampala International University, Kampala, Uganda; 5 Department of Urogynaecology, Mulago Specialized Women and Neonatal Hospital, Kampala, Uganda; 6 Department of Obstetrics, Gynecology and Reproductive Sciences, University of California, San Francisco, San Francisco, CA USA

**Keywords:** Infertility counseling, genital fistula repair, qualitative study, healthcare providers, emotional support.

## Abstract

**Background:**

Infertility counseling for women after genital fistula repair presents unique medical, emotional, and social challenges. This study aims to explore the counseling strategies employed by fistula care providers in Uganda, the challenges they face, and to identify implications for practice.

**Methods:**

This qualitative study involved in-depth interviews with 30 fistula care providers in Uganda. The interviews focused on their experiences, strategies, and challenges in providing infertility counseling to women who had undergone genital fistula repair. The data were analyzed thematically to identify key themes and insights.

**Results:**

The study identified various counseling strategies, including providing emotional support, setting realistic expectations, and referring patients to specialists. Providers have employed individualized approaches, particularly considering patients’ hysterectomy status, with additional counseling sessions for those affected. Cultural attitudes toward infertility influence the counseling process, and financial constraints significantly impact patients’ options. Providers reported emotional challenges in discussing sensitive issues and managing patients’ responses, highlighting a need for better training and support.

**Conclusions:**

Infertility counseling for women after genital fistula repair requires a comprehensive approach addressing medical, emotional, and social dimensions. Our findings highlight significant provider challenges, including the emotional toll of counseling, lack of specific training, and systemic barriers such as patient financial constraints and limited access to specialist care. Enhancing training for fistula care providers in counseling skills and integrating psychosocial support can improve care quality and address these challenges effectively.

## Background

Female genital fistula refers to an abnormal connection between the reproductive tract and adjacent pelvic organs [1]. It can arise from multiple causes, including prolonged or obstructed labor during childbirth, cesarean section, traumatic injuries to pelvic organs, surgical procedures, sexual violence, and genital infections [2, 3]. Globally, an estimated 500,000 or more women live with untreated obstetric fistulas [4]. Additionally, each year, between 50,000 and 100,000 new cases are reported, primarily in sub-Saharan Africa and South Asia [5]. The public health significance of obstetric fistulas, those caused by childbirth, is profound, as they serve as a stark indicator of maternal morbidity and the failure of healthcare systems to provide timely and appropriate care during childbirth [6]. In Uganda, obstetric fistula remains prevalent due to socioeconomic factors such as poverty, early marriage, limited access to emergency obstetric care and skilled birth attendants [7].

Surgical repair remains the cornerstone of treatment, with evidence demonstrating both its effectiveness and cost efficiency [8, 9]. Significant progress has been made in expanding access to obstetric fistula surgery [10] through various targeted national and international efforts [11], and many women achieve successful repairs [8]. However, some women who experience fistula may experience secondary infertility if they have undergone hysterectomy or experienced extensive scarring or infectious complications either during the birth where the fistula developed or fistula repair [12]. Additionally, surgical intervention aimed at repairing the fistula may itself lead to complications such as amenorrhea, intrauterine scarring, biological or physiological dysfunction of the reproductive system, leading to secondary infertility [12, 13].

Infertility is defined as an inability to conceive after one year of regular unprotected sexual activity [14]. Secondary infertility, in contrast to primary infertility, refers to the inability to become pregnant following a prior pregnancy, which may arise from diverse physiological causes, including obstetric fistula and surgical repair [14, 15]. Infertility following fistula repair represents a distinct reproductive and psychosocial concern as the affected women experience secondary infertility not childlessness per se. However, loss of future fertility, loss of reproductive identity can potentially threaten marital security and disrupt anticipated social roles. Research on women from the African sub-continent who have experienced fistula and undergone fistula repair show decreased fertility rates and an increased risk of adverse maternal and neonatal outcomes, such as stillbirth, fistula recurrence, and maternal death, in future pregnancies [13, 15, 16]. In Uganda, these challenges are further compounded among this population by limited access to quality maternal healthcare, social stigma, and economic hardship, which further exacerbate the challenges of fistula and its consequences for affected women.

The psychosocial consequences of infertility are multifaceted and include emotional distress, feelings of shame and guilt, strained relationships with partners and family, social isolation, and mental health issues such as depression and anxiety [16–18].Women in sub-Saharan Africa, in particular, often face social hardships due to infertility, driven by gender and postnatal norms, cultural beliefs, moral concerns, and limited access to specialty infertility care [19].A recent scoping review of fistula-related stigma identified infertility as the predominant intersectional stigma experienced by women with fistula, yet identified very limited fistula stigma interventions [20].While international guidelines on infertility counseling exist [21], and the literature supports the potential for secondary infertility among women with fistula [12], there is no standard practice for counseling women at the time of fistula repair nor specific protocol for supporting women who do face infertility after fistula repair. In Uganda where our research is based, infertility care has only recently been incorporated within the public health system, yet access remains limited [22]. Given the complexity of complications associated with post genital fistula repair, such as infertility and risks in future pregnancies, the limited literature on counseling for infertility among this population, and fertility norms in this context, our study aimed to explore counseling strategies employed by fistula care providers and the challenges they faced in counseling women with infertility concerns after fistula repair in Uganda.

## Methods

### Study design

This study employed a qualitative, exploratory design using in-depth interviews embedded within a larger mixed-methods study that sought to understand women’s experiences of pregnancy and outcomes following female genital fistula repair in Uganda [23]. The present manuscript reports exclusively on the qualitative component of that study and focuses solely on in-depth interviews with fistula care providers. This study focuses on understanding provider counseling practices, including their perspectives on the experiences, strategies, and the unique challenges fistula care providers face when addressing secondary infertility concerns of women who had undergone fistula repair surgery. Qualitative data for this paper were independently analysed and were not integrated with quantitative findings.

### Study setting

The research was conducted at six healthcare facilities in Uganda. These facilities were chosen because of their high volume of fistula repairs and existing clinical collaborations. The participants were from the Eastern Region, Central Region, Northern Region, and Western Region of Uganda.

### Study participants

The participants in the study included fistula care providers, including surgeons, nurses, and other healthcare professionals involved in the diagnosis, treatment, and postoperative care of women with genital fistulas. Providers were selected on the basis of their experience and expertise in managing female genital fistulas of obstetric, iatrogenic, or traumatic etiology. Providers were excluded if they lacked direct experience in treating female genital fistulas or if they were unwilling or unable to provide informed consent for participation in the study. A purposive sample of 30 providers involved in either the clinical or social care of fistula patients were recruited. These individuals were known to the study investigators due to their country-wide engagement in clinical fistula care, research and training.

### Study procedures

Fistula care providers were contacted by phone or in person and invited to participate in in-depth interviews. After providing informed consent, the interviews were scheduled for a time and place convenient for the participants. Most interviews were conducted within consultation or meeting rooms of the healthcare facilities, ensuring confidentiality, privacy and comfort of the participants. The interviews followed a semi -structured interview guide that sought participant perspectives and experiences with infertility post fistula repair, counseling practices, challenges, strategies, and perceived effectiveness. The interviews were audio-recorded with participant consent and lasted for 1–2 h.

### Data analysis

The interview recordings were translated and transcribed verbatim and analyzed via thematic analysis to identify common themes and variations in counseling practices and infertility experiences. The analysis followed a two-stage systematic process. First, the transcripts were reviewed to identify conceptual categories, and initial questions were used as a guide. Deductive codes from literature and interview guides, along with inductive codes emerging from the data, were applied. A codebook detailing each code, criterion, and example was developed. The data were coded via Dedoose software, the potential themes were labeled, and the emerging patterns were interpreted. To minimize potential bias, a semi-structured interview guide was used consistently across interviews. Analysis involved researchers reviewing transcripts and discussing interpretations as a team to refine codes and themes. Data collection and analysis was done by research team members who had no professional relationships with the participants.

### Ethical approvals

Study procedures were reviewed and approved by the University of California San Francisco Institutional Review Board (IRB# 19-27901), the Mulago Hospital Research and Ethics Committee (MHREC# 1674), and Uganda National Council for Science and Technology (HS 2706). All participants provided written confirmation of informed consent. In in-depth interviews with providers, all study participants were instructed to avoid sharing identifiable details, all patient-related information was discussed in anonymized form, and transcripts were de-identified prior to analysis to keep the provider and facility identifying information confidential.

## Results

Thematic analysis of interviews with fistula care providers identified several key themes related to infertility counseling following fistula repair. These themes encompassed when and how counseling was provided, the strategies used to address confirmed and potential infertility, and the challenges providers faced in delivering infertility-related counseling. The results are presented below, beginning with participant demographics and clinical experience, followed by thematic findings illustrated with representative quotes.

### Demographics and clinical experience

The study involved 30 fistula care providers with a median age of 38 years (IQR 32–52). Most participants were female (87%, *n* = 26), while males accounted for 13% (*n* = 4). The professional categories included surgeons (16.7%, *n* = 5), nurses (56.7%, *n* = 17), midwives (20%, *n* = 6), and social workers (6.7%, *n* = 2).

The participants had varying lengths of experience in fistula care: 10% (*n* = 3) had less than 1 year, 3% (*n* = 1) had 1–2 years, 30% (*n* = 9) had 3–5 years, 27% (*n* = 8) had 6–10 years, and 30% (*n* = 9) had more than 10 years. The median number of patients seen per year by these providers was 55 (IQR 30–67). Additionally, 27% (*n* = 8) of the participants had experience as trainers in fistula care, whereas 73% (*n* = 22) did not.

The facilities where the participants worked were diverse. A total of 43.3% (*n* = 13) were from community hospitals or private not-for-profit facilities, 23.3% (*n* = 7) were from specialized public hospitals, 20% (*n* = 6) were from regional referral hospitals, 10% (*n* = 3) were from private not-for-profit hospitals, and 3.3% (*n* = 1) were from a public health center IV.^1^ Most participants were from Eastern Uganda (*n* = 13) or Central Uganda (*n* = 12), with fewer from Western Uganda (*n* = 3) or Northern Uganda (*n* = 2).

### Timeline and context of counseling

Infertility counseling for women following surgical repair occurred both at the time of surgery during which patients stay at the fistula repair facility for approximately two weeks, and during follow-up surgical review visits. This structure allowed for the topic of confirmed or potential infertility to be brought up multiple times. Providers shared that this was helpful because it helped orient patients to known expectations (i.e., women who had undergone hysterectomy during the childbirth that resulted in fistula due to complications) versus unknown potential of post-repair infertility. It also helped them set realistic expectations and prepare patients emotionally for possible outcomes. Providers acknowledged the importance of future childbearing following fistula repair among many of their patients, particularly those who were younger or who had fewer children, and other counseling conversations centered around provider guidance regarding recommended timeframes for abstaining from penetrative sexual activity following fistula repair and the importance of delaying immediate post-repair pregnancy to optimize repair outcomes. These topics were heavily focused on at the time of fistula repair, whereas during surgical follow-up visits, providers described conducting assessments to evaluate level of patient physical recovery, provide continuing emotional support, suggest coping strategies per specific patient need, and provide referrals for any further medical evaluation required.

### Providing counseling strategies for infertility concerns following genital fistula repair

Providers described very different counseling strategies for individuals who brought up infertility concerns at the time of fistula repair or follow-up care based on their hysterectomy status, which confirmed infertility, and was known at the time of fistula repair. For individuals who had undergone hysterectomy during the birth that resulted in fistula, these strategies were largely focused on helping patients understand and accept the consequences of the complications they experienced, educating them on alternative options to have children or for those who had existing children, helping them to consider the importance of focusing on those children, finding solace in religion, and focusing on economic stability and quality of life. For those who had concerns about potential infertility or who were followed-up with fistula care providers after not becoming pregnant following the fistula repair, providers described referring for specialty care, acknowledging the emotional impact of potential infertility, engaging male partners, and finding solace in religion. These strategies are described in more detail below:

#### Patients with confirmed hysterectomy

##### Emotional support and facilitation of acceptance

The most common counseling strategy reported by fistula providers for women who had undergone hysterectomy was to help patients understand and accept the need for this surgery during the fistula causing birth, and appreciate the specific consequences of hysterectomy, providing emotional support throughout. Providers described ongoing counseling sessions as opportunities to provide women with the necessary resources to help them cope with confirmed infertility and processing their experiences and concerns by emphasizing the medical circumstances that led to the hysterectomy and facilitating acceptance of their new reality. Providers described educating women that due to the removal of their uterus, conception would no longer be possible, and menstruation would no longer occur. This information, delivered sensitively, was considered to help facilitate emotional adjustment and acceptance of their reproductive loss. Providers shared the following narratives of how they might support a patient whose uterus was removed due to rupture during labor:

*“I try to find out about the operation that had been done on them because a good number of patients aren’t sure what they did to them. For those whose uterus was removed*,* although some of them know*,* I tell them that you had an emergency while giving birth and your uterus ruptured beyond repair or was damaged and had to be removed to save your life. With this*,* you won’t be able to conceive or have your periods.” -Social worker Eastern Region.*

*“ We let them go through the grieving process and after they have calmed down*,* some of them respond by saying*,* “I have enough kids*,* but since my husband didn’t allow family planning*,* I had to try again.” So*,* we talk to them one day at a time while reminding them that it doesn’t make them less of human beings because they can’t have children. We encourage them to keep calling and we make time for more sessions with them whenever they feel like talking to us. … Of course*,* they withdraw emotionally and there’s low self-esteem that comes with it. So*,* we ask them about their support system because it is very important and most of them decide to go back to their parents so that they can feel rehabilitated while they’re with their mothers. We mostly let them speak up.” – Social Worker*,* Central Region*.

Providers described the health education required and how they clearly communicated the implications of uterus removal, emphasizing that the uterus is essential for childbearing, and without it, conception is impossible. This straightforward and factual communication was intended to help women understand and accepted of the consequences of the procedure, and its impact on reproductive capabilities.

*“[…]We tell them that for someone to get pregnant*,* you need to have a uterus; however*,* you lost yours during the delivery*,* and you’ll never become pregnant. That is what we normally tell them”.- Midwife*,* Central Region*.

##### Alternate options to have children

Some providers reported that they encouraged women to explore alternative paths to parenthood, such as adoption or surrogacy. They described educating patients about these options, empowering them with information and resources tailored to individual needs and aiming to offer hope and practical solutions for women wishing to have children despite their infertility; however, they also acknowledged the high cost of these procedures and relative inaccessibility for this patient population due to their socioeconomic status. These were shared with patients, as follows:

*“[…. ]You advise on other methods that she can use to have children as a couple like adoption*,* and IVF. However*,* these methods are very expensive for them (as many fistula patients are of low socioeconomic status). The husband can choose to get other women to have children. We make them accept the situation and then they can choose a way forward.”- Eastern Midwife Region*.

*“[…]This is now how things are - naturally*,* you cannot conceive. But these days*,* there are ways of conceiving. These days*,* some women carry pregnancies for other people. So*,* if you can afford*,* in the future*,* for instance when you are married and you want a child.”- Nurse Central Region*.

##### Focus on raising existing children

Providers acknowledged the importance to their patients of achieving their desired family sizes, but in cases of secondary infertility, they counseled women with existing children to focus on raising these children. Providers emphasized that, despite the inability to have more children, these women could still find purpose and fulfillment by concentrating on nurturing and improving the quality of life of the children they already have. Some providers further wove in the perspective that these women were lucky to have other children, as some infertile women with fistula had none, and further emphasized the significant impact of the fistula from childbirth, as seen in the following quotations:

*“[…]Therefore*,* you counsel her such that she accepts that despite what happened*,* she can still live and raise her children because some women don’t have a child. You counsel her until she accepts that much as that is what has happened*,* the good thing is that she is not leaking urine anymore*,* ‘you will still do your normal activities and raise your children*,*’ we tell her.”- Midwife Central Region*.

*“You have 5 children*,* and this [fistula] has affected you so much; please concentrate on raising the children that you already have so that you can improve the quality of your life.”- Midwife Eastern Region*.

##### Personal empowerment and vocational rehabilitation

Some providers viewed women without a uterus as having a disability and encouraged them to pursue further education, vocational training, and financial empowerment activities as part of their fistula recovery. They reported facilitating access to resources such as school fees, job opportunities, and capital for starting small businesses. These efforts were aimed at providing tools for personal empowerment and vocational rehabilitation, providers aimed to support women’s holistic well-being and independence post-surgery. In doing this, providers also acknowledged the potential repercussions of not being able to have children on women’s relationships and the importance of ensuring she was financially stable on her own and did not have to rely on a partner.

*“[…] Most of them don’t even know what’s going on so*,* we let them go through the grieving process. Some of them want to go back to school*,* and their parents are willing to pay their school fees. We also tell them to focus their energy somewhere else like a job.” Social worker central region.*

*“[…] Most of the time*,* we have had groups that help women who have that disability. We treat her like she’s disabled. There is a group of women that would come….let’s say we put them in a group of disabled people so*,* they would give her something to do*,* and offer her with capital to start a business to distract her and she doesn’t have to ask anyone for anything because she doesn’t have money. In this case*,* they are given tailoring machines*,* saloons (hair saloons)*,* and basic needs. This is intended to make sure that they take care of themselves for … even when there is a man that is interested in her*,* he can love her as she is (without producing children). The problem is the fact that she needs to ask for help from the man who will say that he is not getting anything from her*,* so we usually send them [to these programs].” Nurse*,* Central Region.*

##### Sexual activity beyond conception

Providers emphasized the importance of resuming sexual activity safely, advising women to wait at least three months after fistula repair before resuming sexual activity to allow adequate time for healing. Many of these conversations focused on the importance of sexual intimacy beyond reproduction, and providers reassured patients that they could continue to safely experience sexual intimacy and maintain emotional and physical closeness with their partners, promoting a high quality relationship, even though they could no longer conceive.

*“[….] counsel her until she accepts that much as that is what has happened*,* the good thing is that she is not leaking urine anymore; “you will still do your normal activities and raise your children*,*” we tell her. Secondly*,* she would know that she can still have intercourse with her husband but would not be able to give him a child. So that is how we usually counsel them. “-Midwife Central Region.*

*“[….]When we go to them*,* we also emphasize that they lost their uteruses and they won’t have any children however*,* they can still have sexual activity with their husbands.” -Midwife*,* Central Region*.

##### Spiritual guidance

Providers shared how they recommended integrating spiritual guidance into their counseling sessions, ensuring that it was in keeping with the patient’s preferences and the cultural context. They encouraged a habit of prayer and trust in God, often describing the situation as an aspect of a divine plan. This was intended to foster emotional resilience, acceptance, and provide support during the recovery process.

*“[…. ]Personally*,* I do a lot of preaching to them because the Bible tells us to always thank God. ‘Yes*,* you lost your child and uterus*,* but everything happens for a reason; you never know it could have been your life*,* but God knew you even before you were born.’ Of course*,* some react and get emotional and start crying but after all of that*,* we tell them that we should plan on how to move on with life. If the uterus is lost*,* now we need to learn to live without it. Of course*,* they will cry but we can’t change what has happened so*,* we need to move on.”- Nurse*, *Eastern Region.*

*“[…. ]the other advice I always give is urging them to pray more because you are counseled more when you pray. The mother also needs to understand what happened to her and understand that they need to put God first. If you understand that what happened was not your own making*,* you do not have any power over it*,* and you actually do not know what your life plan is; you need to pray and take counsel in GOD.”—Social worker*,* Central Region*.

#### For patients with uncertain causes of infertility

##### Referral to gynecologists for further evaluation and management

For patients whose infertility could not be explained by their fistula condition or clinical history, providers described referring to gynecologists for further evaluation and management by individuals with the appropriate expertise. They emphasized the importance of addressing potential infections, hormonal factors, and other underlying causes of infertility. By involving specialists, providers intend to ensure comprehensive care and appropriate treatment for these patients. Many providers discussed being able to refer their patients to this expertise within the same facility, which facilitated this care access, yet for others, patient accessibility presented a greater challenge.

*“[…. ]we refer them to a gynecology clinic because some of them could be having infections*,* and some of them it is due to hormonal factors that they fail to conceive until we refer them to the gynecology clinic.” -Midwife*,* Central Region*.

*“[…]We refer them to gynecologists to find out other causes of mothers not conceiving other than vvf. Failure of mothers not being able to conceive is not attributed to only vvf; there are other causes.” Nurse Eastern Region*.

##### Emotional support

Providers described the counseling sessions as focused predominantly on acknowledging patients’ substantial emotional suffering due to infertility concerns and discussed their role as fistula providers without substantial infertility expertise as supporting women both to access necessary care and to accept the uncertainty of their situation. Providers mentioned that they offered comfort and encouraged patience, while also referring patients to medical facilities for further evaluation of infertility or assisted reproductive technology services as appropriate. This holistic approach was intended to address both the emotional and medical needs of patients.

*“[…] As a care provider and nurse*,* I really can’t do much about it. Maybe I can refer them to a gynaecologist that has more knowledge about these issues. But as a nurse*,* I just need to comfort and ask them to be patient.”- Nurse*,* Eastern Region.*

##### Involvement of male partners

Providers also reported the importance of involving male partners in the evaluation process, recognizing that the infertility issues could sometimes be attributed to them. By inviting partners for fertility assessments, providers ensured a comprehensive approach to addressing infertility, acknowledging that the problem might not always lie with the woman.

*“[…]We always want the gynecologist to attend to them because [they] will know what needs to be prescribed…. Sometimes [the gynecologist] invites their partners to make sure he is not the one with the problem because at times the first child was the only child that he could father.”- Nurse*,* Central Region*.

### Challenges faced by providers in providing effective counseling on infertility concerns following female genital fistula repair

Providers reported that they faced a range of challenges to improve the effectiveness of counseling to women dealing with infertility concerns following female genital fistula repair. These challenges spanned from communication difficulties and emotional tolls to logistical barriers, as detailed below:

#### Sensitive communication

Providers stated that they faced significant challenges in effectively communicating sensitive information regarding prior surgeries and fertility implications, infertility consequences, and alternative options for having a child. Many counsellors said that they struggled while discussing the implications of uterus removal due to the large emotional impact and the associated social stigma of not being able to bear children. They also acknowledged important practical limitations to the accessibility of alternative options for children, particularly given the low socioeconomic status of many women with history of fistula, with multiple providers acknowledging the cost of these services would be prohibitive.

*“[…]For those that have had children before*,* we just encourage them to rely on their children and if a mother doesn’t have any children*,* it is troublesome. For example*,* the client I told you about earlier who got 2 miscarriages and later got a hysterectomy*,* it was so hard and it is still giving her a lot of social stigma. Currently*,* she’s undergoing counselling and I can’t stop talking to her all the time. It is really not easy. “-Nurse Northern Region.*

#### Emotional impact

Per provider respondents, counseling women with post repair infertility was not only emotional for patients but also took a notable emotional toll on the providers themselves. They reported that their own emotional burden intensified when clients reacted with tears, anger, or left sessions abruptly. Counsellors also mentioned that they frequently encountered intense emotional responses ranging from denial to despair, which made their work challenging.

*[…]It was not easy. It was a series of counseling because it cannot be done in one day. I give her a series of counseling for her to finally accept it*,* and she later understood that she can’t have children.” – Nurse*,* Eastern Region*.

*“[…] I pray that we don’t get any of these [post-repair infertility] cases because it is a very hard thing to deal with*,* as they come back wanting to have surgeries. For example*,* we had a patient who had very many repairs … and some experts said that she won’t be able to conceive again.” –Nurse*,* Eastern Region*.

#### Lack of adequate skills

Many of the previous challenges that the providers described experiencing with communicating sensitive information, especially those related to informing women that they had previously had a hysterectomy and would no longer be able to become pregnant, were attributed to limited experience and perceived deficiencies in counseling skills. Some providers emphasized the need for support from psychosocial specialists to address the complex emotional needs of these women and the need for nuanced communication and deeper emotional engagement.

*“[…] I explain to them what happened. I always give a small package because when they hear that the uterus has been removed*,* we involve the psycho-social counsellors to come and handle that case. I do not have answers or good counseling skills” – Nurse*,* Western Region.*

#### Financial constraints

Providers’ discussions around alternative strategies for conceiving were often complicated by their appreciation of women’s financial constraints. Providers expressed feeling ill-equipped to address these issues, as they lack of control over financial circumstances influencing women’s ability to pursue such options combined with few publicly-available resources for this type of care.

*“[…]We talk about it however*,* they are poor; currently*,* women without uteruses can have children; however*,* it is an expensive procedure*,* and as you know*,* our clients are financially constrained. You can suggest this idea to her and she tells you*,* “I can’t afford it.” Midwife*,* Central Region*.

#### Confidentiality concerns

Providers noted that many women with infertility, influenced by societal stigma around reproductive loss, often requested that their condition to be kept for confidential from spouses or family members. This presented a challenge for counsellors who were committed to upholding patient confidentiality while also being concerned about the potential negative impact this lack of disclosure might have on their relationships and family support systems.

*“[…] A large number of clients requested that their partners never determine about it and because of patient confidentiality*,* we do not disclose. [Sharing this information with their partner] is their decision to make*,* and when they are ready*,* they will disclose it to them. “ – Social worker*,* Central Region*.

#### Partner resistance

Providers highlighted the important role of partner support and understanding in helping women accept infertility or potential infertility. They noted that partner refusal to engage in discussion with the woman or the provider, it can significantly undermine the effectiveness of counseling and support. Providers believed that partner understanding of the reason for infertility was paramount to their own acceptance of the situation and their support of the women. Yet, providers observed partner’s unwillingness to acknowledge or understand the consequences of infertility.

*“[…].That is challenging*,* but we need both partners for this counseling because*,* in this case*,* the most important thing is for the partner to understand what caused this mother to lose the uterus and what happened along the way. If the partner understands this*,* there will be no problem; however*,* the challenge is if they do not understand.” –Social worker*,* Central Region*.

These attitudes and expectations are likely responsible for women’s lack of interest in disclosing infertility status to partners, as described above.

## Discussion

This exploratory study reveals an important need for effective provider counseling around confirmed and potential infertility among women undergoing genital fistula repair. The findings highlight a concerning lack of information and understanding among patients regarding their fertility status, including around prior birth complications and their impacts on future reproductive status, and the need for fistula repair providers to competently meet these needs. Providers counselled patients using a variety of strategies including providing emotional support, setting realistic expectations, and sending appropriate referrals to specialists for further evaluation and management. However, providers in our study reported a wide variety of challenges counseling this patient population due to lack of training, the emotional nature and anticipated stigma of the topic, and lack of accessible patient resources, limiting their ability to provide comprehensive infertility-related counseling, particularly for women whose infertility status was known, and causing substantial provider stress. As infertility following fistula repair represents a form of secondary infertility, the provider counseling addressed concerns related to loss of future fertility in the context of irreversible reproductive loss. Provider narratives identified key areas for improvements including provider education and standard protocols to improve both provider competency and patient experience.

The provider counseling strategies described are theoretically consistent with an evidence-based collaborative mental health care model which emphasizes coordinated care between primary care providers, mental health specialists and community resources [24]. The counseling strategies described in this study, though sometimes delivered in a single session, reflect key elements of this model and have been proven to be effective even with a single session in some prior studies for mental health concerns [25]. However, the practicalities of implementation appeared to be significantly limited by contextual factors and resources and lack of follow-up.

Building on providers’ narratives, efforts to expand quality infertility counseling following this model should include extending appropriate health education counseling into the birth setting, ensuring that women with hysterectomy are appropriately informed and counseled around the emergency procedures they underwent and their implications. Providers mentioned challenges related to patients’ understanding of reproductive procedures and outcomes, attributing these difficulties to limited health literacy, variability in patient–provider communication, and the emergency nature of obstetric complications leading to hysterectomy. Similar findings have been reported in Ugandan urban teaching hospitals, where patients and family members described variability in their understanding of emergency surgical consent and the implications of procedures performed [26, 27]. This highlights the structural challenges to achieving fully informed consent in emergency obstetric contexts and underscore the importance of strengthening postoperative communication to ensure women understand the reproductive consequences of hysterectomy [28]. 

Given the different potential causes of infertility and infertility concerns, individualized approaches or approaches using key stratifying factors (e.g., hysterectomy status) are likely to be most successful for this patient population. Provider narratives in our study documented emotional support as a central component of counseling for all women experiencing infertility, with a demonstrated need for additional counseling sessions among those who have undergone a hysterectomy [29, 30]. In addition to addressing emotional needs, providers guided patients in accepting their infertility and recommended exploring alternative paths to parenthood, such as adoption or surrogacy. This approach underscores the proactive and comprehensive nature of the support provided by care providers; however, as was noted by the providers in our study and consistent with the literature, practical access to these alternative paths is limited within this setting and in particular, among this population which is generally of lower socioeconomic status [22, 31].

For patients with unexplained infertility, the predominant strategy for fistula providers is to refer patients for specialty fertility clinics for further evaluation and treatment. This strategy sought to ensure that patients received comprehensive care that addressed both their medical and psychological needs. However, the specific cultural and social context significantly impacts the counseling strategies and challenges faced by providers. Societal attitudes toward infertility and the loss of reproductive capacity often exacerbate the emotional distress experienced by women. Providers must navigate these cultural norms while providing support, advocate for assisted reproductive technologies, and encourage patients to explore alternative aspects of their lives, such as vocational training and financial empowerment [32], to improve their quality of life and overcome potential partner support challenges due to infertility.

Our findings are consistent with other research which has identified substantial impacts to infertility in the East African setting, including possible quality of life and relationship impacts [33]. For example, among women who underwent emergency hysterectomy as definitive treatment for hemorrhage, Pilli et al. corroborated the significant concerns around infertility stigma and partner abandonment identified in our study [33], also reflected in the broader fistula stigma literature [20]. In our research, stigma was an important consideration for infertility disclosure and may ultimately influence women’s seeking and access of infertility care, as is seen elsewhere [34].

Most providers reported significant emotional challenges associated with counseling, including difficulties communicating sensitive issues including hysterectomy and managing patients’ intense emotional responses. This underscores the considerable emotional burden on providers and the need for support systems to assist them in this demanding role. The burden is compounded by the fact that many providers have received only training in fistula care and lack adequate preparation for handling sensitive conversations and managing intense emotions, and the limitations to accessibility of mental health care [35]. Additionally, the financial constraints reported by patients, which limit their ability to consider alternatives such as surrogacy or adoption, add another layer of complexity to the counseling process and providers’ ability to provide tangible recommendations. Providers are often not equipped to address these financial challenges, further complicating their efforts to support patients effectively, and increasing their likelihood of burnout due to this stress and inability to provide optimal care [36].

### Strengths and limitations

This formative study contributes to the limited literature on this topic. The findings, derived from interviews with 30 fistula care providers in Uganda, may not be applicable to a global context. However, given that this is a qualitative study, the primary aim was to explore phenomena rather than to generalize the findings. The study’s strengths include diversity of providers from various professional roles and facilities, which allowed us to capture a wide scope of perspectives, and the use of a systematic analytic approach to ensure transparency and rigor in our findings. The reliance on self-reported data from in-depth interviews of healthcare providers may not reflect actual counseling behaviors due to potential recall and social desirability biases and may affect the validity of the results. Provider gender was not explicitly examined in this study and did not emerge as a distinct analytic theme; however, the sample was predominantly female, which may have influenced counseling dynamics and patient disclosure. Moreover, the study focused solely on the perspectives of fistula care providers and did not include direct input from women who underwent counseling, which could have offered a more comprehensive understanding of the counseling process, nor did it include the perspectives of infertility providers. This study did not specifically examine differences in counseling practices by rural–urban location, facility type, or patient socioeconomic or educational status, which may have shaped the time available for counseling, the depth of infertility discussions, and patient understanding and engagement.

### Implications for practice

The study’s findings underscore several key implications for practice. Firstly, the variability in reported counseling practices reflects differences in competencies, personal approaches and available resources among providers, making consistent counseling practices challenging within the setting. Additionally, several providers referred to a lack of standard guidelines or facility protocols on this counseling topic, indicating a gap in care standards that should be resolved. Secondly, there is a clear need to enhance training for fistula care providers in counseling skills, with a focus on handling sensitive information, offering emotional support, and managing intense emotions. Integrating psychosocial counselors into the care team could improve the management of complex cases involving significant emotional and psychological needs. Furthermore, addressing structural barriers is crucial. This includes addressing financial constraints, improving access to specialist services, promoting community awareness, and creating opportunities for vocational rehabilitation. Such measures are essential for providing comprehensive and effective counseling to women dealing with infertility following fistula repair. 

## Conclusion

Infertility counseling for women post-genital fistula repair is a complex and emotional process that requires a multifaceted approach.Our study has identified that fistula care providers in Uganda employ various counseling strategies, including providing emotional support, setting realistic expectations about outcomes, and referring patients to specialists. By addressing the unique medical, emotional, and social challenges faced by these women, fistula care providers play a crucial role in helping them navigate their infertility journey. The study findings also highlight the challenges faced by fistula care providers, such as emotional burden of work, limited training in psychosocial counseling approaches, and structural barriers like financial constraints and social stigma. Enhanced training, the integration of psychosocial support, and addressing structural barriers are essential steps toward improving the quality of care and support for these patients.

Future research should focus on developing and evaluating interventions aimed at improving the counseling skills of fistula care providers. Additionally, there is a need for research on the feasibility and effectiveness of peer support interventions in low- and middle-income countries to inform best practices in the context of limited healthcare resources.

## Data Availability

The availability of data and materials will be considered on a case-by-case basis due to the qualitative nature and potential for identifiability. Specific requests for access to the data or materials used in this study may be made to the corresponding author.

## Footnotes

1 In the Ugandan context, a Public Health Center IV (HC IV) is a sub-district level health facility serving approximately 100,000 people and offering a range of services including preventive and promotive care, outpatient and inpatient care, maternity services, emergency surgery, and blood transfusions, in addition to the services provided by lower-level centers.
